# Supplementary material for: Potential Correlation between Microbial Diversity and Volatile Flavor Compounds in Different Types of Korean Dry-Fermented Sausages
Source: Foods. 2022 Oct 12;11(20):3182. doi: 10.3390/foods11203182 (PMC9602160; doi:10.3390/foods11203182)
Supplement: Supplementary file 1 [file foods-11-03182-s001.zip › foods-1918301-supplementary.pdf]

**Table S1.** Relative abundance (%) of the fungi community proportions at species levels in dry-fermented sausage samples from four different regions of Korea (cut off: ≥ 0.1% abundance).

| Species | A | B | C | D |
|---|---|---|---|---|
| *Aspergillus ruber* | 0.0 | 0.0 | 0.0 (17) | 3.9 (3,249) |
| *Candida metapsilosis* | 0.4 (165) | 0.0 | 0.0 | 0.0 |
| *Candida parapsilosis* | 0.0 | 1.1 (767) | 0.0 (31) | 0.0 |
| *Candida smithsonii* | 4.4 (1,912) | 0.5 (331) | 0.0 | 0.0 (37) |
| *Cutaneotrichosporon curvatus* | 0.0 | 0.0 | 0.0 (18) | 0.0 |
| *Cutaneotrichosporon moniliiforme* | 0.0 | 0.0 (28) | 0.0 | 0.0 |
| *Cyberlindnera jadinii* | 0.9 (395) | 0.0 | 0.0 | 0.0 |
| *Debaryomyces prosopidis* | 0.1 (44) | 5.0 (3,473) | 0.7 (599) | 92.4 (77,167) |
| *Hannaella sinensis* | 0.0 | 0.0 | 1.5 (1,345) | 0.0 |
| *Mucor circinelloides* | 0.0 | 0.0 | 0.0 | 1.4 (1,182) |
| *Penicillium bialowiezense* | 0.0 | 0.0 | 0.0 (16) | 0.0 |
| *Penicillium neocrassum* | 0.0 | 0.0 | 0.1 (97) | 0.0 |
| *Penicillium nalgiovense* | 76.0 (33,343) | 93.3 (64, 619) | 96.8 (84,388) | 1.6 (1,372) |
| *Penicillium sumatraense* | 0.0 | 0.0 | 0.0 (23) | 0.0 |
| *Pichia fermentans* | 0.0 | 0.0 (23) | 0.0 | 0.0 |
| *Plectosphaerella cucumerina* | 0.0 | 0.0 | 0.0 (6) | 0.0 |
| *Rhodotorula mucilaginosa* | 0.0 | 0.0 | 0.0 | 0.6 (522) |
| *Saccharomyces arboricola* | 0.3 (150) | 0.0 | 0.0 | 0.0 |
| *Sporobolomyces carnicolor* | 0.0 | 0.0 | 0.7 (646) | 0.0 |
| *Tausonia pullulans* | 0.1 (45) | 0.0 | 0.0 | 0.0 |
| *Wickerhamomyces anomalus* | 17.8 (7,802) | 0.0 | 0.0 | 0.0 |
